# Exploring Nanotechnology as a Strategy to Circumvent Antimicrobial Resistance in Bone and Joint Infections

**DOI:** 10.1021/acsomega.3c01225

**Published:** 2023-04-27

**Authors:** Phumzile P. Skosana, Steward Mudenda, Patrick H. Demana, Bwalya A. Witika

**Affiliations:** † Department of Clinical Pharmacy, School of Pharmacy, Sefako Makgatho Health Sciences University, Pretoria 0208, South Africa; ‡ Department of Pharmacy, School of Health Sciences, University of Zambia, Lusaka 10101, Zambia; § Department of Pharmaceutical Sciences, School of Pharmacy, Sefako Makgatho Health Sciences University, Pretoria 0208, South Africa

## Abstract

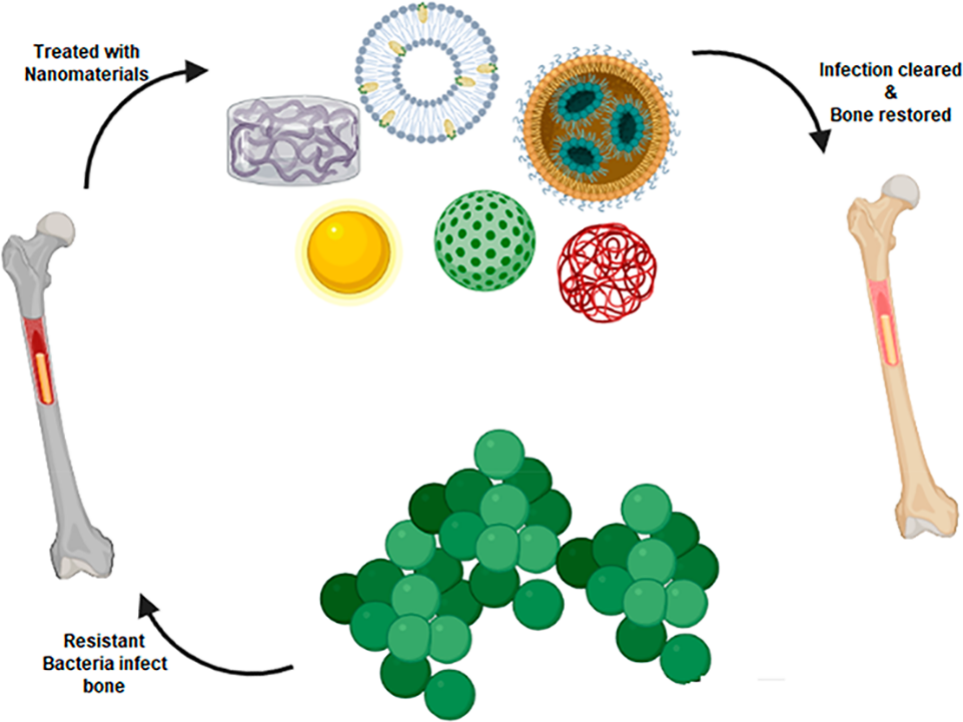

Bone and joint infections (BJIs) are difficult to treat, necessitating antimicrobial therapy at high doses for an extended period of time, in some cases different from our local guidelines. As a consequence of the rise in antimicrobial-resistant organisms, drugs that were previously reserved for last-line defense are now being used as first line treatment, and the pill burden and adverse effects on patients are leading to nonadherence, encouraging antimicrobial resistance (AMR) to these last-resort medicines. Nanodrug delivery is the field of pharmaceutical sciences and drug delivery which combines nanotechnology with chemotherapy and/or diagnostics to improve treatment and diagnostic outcomes by targeting specific cells or tissues affected. Delivery systems based on lipids, polymers, metals, and sugars have been used in an attempt to provide a way around AMR. This technology has the potential to improve drug delivery by targeting the site of infection and using the appropriate amount of antibiotics to treat BJIs caused by highly resistant organisms. This Review aims to provide an in-depth examination of various nanodrug delivery systems used to target the causative agents in BJI.

## 1 Introduction

Antimicrobials have been essential to modern healthcare since their introduction into medicine in the 1940s.^1,2^ Patients receiving chemotherapy,^3,4^ those with chronic illnesses like diabetes mellitus, end-stage renal disease, and rheumatoid arthritis, and those who have undergone complex surgeries like organ transplants, joint replacements, or cardiac surgery have all been treated successfully and have had prevention from infections.^3^

Many surgical procedures and immunosuppressive treatments rely on antibiotic prophylaxis and the ability to treat infectious complications.^4^ Antimicrobials have been repeatedly shown to considerably lower morbidity and mortality from infections.^5^ Antibiotic-resistant pathogens, on the other hand, have emerged and spread throughout human and animal populations worldwide.^6−8^

The occurrence of antimicrobial resistance (AMR) in humans, animals, and the environment is a natural phenomenon; however, due to the overuse and misuse of antimicrobials in human healthcare, animals, and the environment, its evolution has been expedited.^9−13^ AMR is one of the public health issues that has contributed to increased morbidity and mortality around the world.^14−16^ According to World Bank research, AMR could cost low-income countries more than 5% of their GDP and push 28 million people into poverty by 2050, primarily in the developing world.^17,18^ It has been reported to be a global threat to human and animal life, and if not addressed, it will continue to cause harm.^15,19^ Due to this problem affecting both humans and animals, a “One Health” approach is required to address it, which entails the collaborative efforts of multiple disciplines working locally, nationally, and globally to achieve optimal health for people, animals, and our environment.^8,20−24^ As a result, to successfully address this issue, the factors that contribute to it must be identified and addressed.^25−27^

The inappropriate use of antibiotics in humans and animals has contributed to the development and worsening of AMR.^28^ One of the major factors contributing to AMR in humans has been reported to be the ease with which antimicrobials can be obtained without a prescription.^29−31^ Additionally, irrational prescribing of antibiotics has made this phenomenon worse.^25,32−38^ AMR has also been linked to irrational dispensing practices and easy access to antibiotics for use in animals without a prescription.^39−42^ Evidence has also shown that healthcare professionals with this responsibility have missed doses when administering antibiotics, which has been linked to the development of AMR.^39,43−45^ As a result, patient noncompliance with antimicrobial therapy has also contributed to the development of AMR. Furthermore, a lack of patient education on antimicrobial use (AMU) and AMR is another factor that contributes to inappropriate AMU and, eventually, may lead to the development of AMR.^7,25,46^

Strategies to address the issue have been put forth in light of the growth of AMR. Antimicrobial stewardship (AMS) programs have been shown to lower AMR in settings where they have been successfully implemented.^47−49^ The rational prescribing, dispensing, administration, and consumption of antimicrobials are all promoted by AMS.^50−53^ It has been reported that educational initiatives designed to raise healthcare workers’ awareness of AMR are successful in addressing this issue.^46,54,55^

When there is resistance to the commonly prescribed antibiotics, pressure on prescribers increases due to the recent decline in the development of new antimicrobials.^56−58^ This necessitates the development of novel antibiotics as a remedy for AMR.^58−61^ Additionally, there is convincing evidence that alternative therapies can help decrease AMU and AMR.^62−65^ Vaccines are essential for lowering transmission, spread, and severity of disease, which leads to a reduction in AMU and AMR.^66,67^

In this paper, we propose that the use of nanodrug delivery systems may be the solution to tackle highly resistant organisms causing bone and joint infections (BJIs) In this paper, articles were stratified based on nanotechnological approaches to treat BJIs that are caused by highly resistant bacteria.

## 2 Bone and Joint Infections

Bone and joint infections include septic arthritis, prosthetic joint infections, osteomyelitis, spinal infections (discitis, vertebral osteomyelitis, and epidural abscess), and diabetic foot osteomyelitis.^68^ They often cause chronic pain and dysmobility in patients, which contributes considerably to the burden of disease, especially in the elderly. These infections can be caused by a variety of microorganisms, including bacteria, viruses, and fungi, and can occur in any.

The incidence of septic arthritis in developed countries, including the US, is estimated at six per 100,000 population per year, which is estimated to be higher in developing countries.^69^ Males are twice as likely to be affected than females, and pediatric patients account for more than 50% of cases of acute hematogenous osteomyelitis, the most common condition in BJIs^70^

Bone and joint infections can cause a range of signs and symptoms with the common ones being pain and tenderness in the affected area, swelling around the joint or bone, stiffness and limited range of motion in the joint, and many more. These symptoms can vary depending on a number of factors like age, severity, and other comorbidities.^68^

It is worth noting that the symptoms of bone and joint infections can vary depending on the age of the patient and other host factors like the underlying health conditions, the severity of the infection, and the type of microorganism. If not treated accordingly, they may leave patients with a lasting disability due to high rates of recurrence and may even cause death; therefore, timely diagnosis and intervention are vital.^71^

## 3 Antimicrobial Use in Bone and Joint Infections

Treatment of BJI is complicated and requires a coordinated multidisciplinary approach in addition to appropriate medicines.^72^ Compared to soft tissue infections, the penetration of antimicrobial agents into the bones necessitates high dosages.^73^ Osteomyelitis and septic arthritis require antimicrobial treatment for 4–6 and 3–4 weeks, respectively, while prosthetic joint infections involving retained implants, hardware, or prostheses require treatment for 3 to 6 months and sometimes longer.^74^ The selection of an antibiotic to successfully treat the infection is influenced by a variety of factors, such as the bacteria’s susceptibility to antibiotics, the antimicrobial’s capacity to penetrate bone and joint tissue, oral bioavailability, and cost.^75^ Monitoring for toxicity, drug interactions with concurrent prescription drugs, adherence, and tolerability are additional considerations.^76^

Multidrug resistant (MDR) methicillin-resistant *S. aureus* (MRSA) and other antibiotic-resistant organisms for these conditions are on the rise. Only after obtaining culture results, testing for susceptibility to the microorganism, and taking into account the degree of bone penetration can definitive therapy be started.^77^ Penicillins and cephalosporins are the preferred drug class for these conditions because numerous studies and organizations support the use of empiric therapy. A summary of some of the most dominant causative organisms and which antibiotic can be used in those infections is provided in Table 1.

**Table 1:** Summary of the Treatment Regimen against Bacteria That Cause BJI

microorganism	antibiotics	ref
*Methicillin-susceptible S. aureus (MSSA)*	Oxacillin, Cefazolin, Rifampin, Amoxicillin/Clavulanic Acid, Ciprofloxacin, Flucloxacillin, Clindamycin, Ceftriaxone	(73, 75, 78, 79)
*Methicillin-resistant S. aureus (MRSA)*	Teicoplanin, Vancomycin, Rifampin, Co-trimoxazole, Minocycline, Linezolid, Daptomycin	(78)
*Streptococcus spp.*	Amoxicillin, Levofloxacin, Ceftriaxone, Clindamycin	(78)
*Enterobacteriaceae*	Ciprofloxacin, Levofloxacin, Ceftriaxone	(78)
*P. aeruginosa*	Cefepime, Ceftazidime, Ciprofloxacin, Levofloxacin, Piperacillin/Tazobactam, Meropenem, Imipenem	
With biofilm association	Rifampicin, Ciprofloxacin, Levofloxacin, Clindamycin, Linezolid	(73)

### 3.1 Mechanisms of Microbial Infiltration in the Bone and Joints

Antimicrobial resistance (AMR) occurs when microorganisms including bacteria, viruses, fungi, and parasites change over time and no longer respond to medicines that once hindered their multiplication, making infections harder to treat and increasing the risk of disease spread, severe illness, and death.^15^

**Figure 1:**
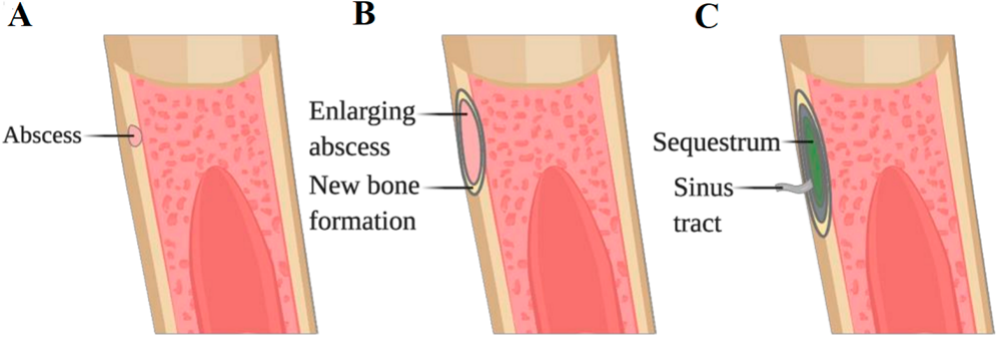
Summary of the progression of osteomyelitis. An abscess develops from a localized infection that constricts the blood flow to the area (A), resulting in an avascular region of necrotic bone tissue called the sequestrum (B), followed by the development of new bone surrounding the sequestrum, termed the involucrum, which may also have a sinus tract through which purulence can escape (C) [Reprinted with permission from ref (81). Copyright 2020 MDPI].

The bone has a low susceptibility to infections, but when a microorganism enters the bone marrow cavity, an infection can develop^80^ as depicted in Figure 1.

Subsequent to bone infiltration, these invasive bacteria produce adhesins for proteins, collagen, laminin, and fibronectin, which enables them to adhere to cartilage.^815^ Acute inflammation occurs, during which phagocytes attempt to suppress these microorganisms, resulting in the production of toxic radicals and the secretion of proteolytic enzymes that lyse the tissue surrounding the bone.^815^ This inflammatory response forms pus (a protein-rich discharge that includes bacteria, tissue fragments, and dead phagocytes), which leaks into vascular channels as a result of inflammation, increasing intraosseous pressure and impairing blood flow.^82^ Lack of blood supply to this area results in the death of bone tissue and the separation of hypoperfused bone fragments known as sequestrum.^815^ Some microorganisms form an impermeable biofilm around them that protects them from the host’s defense mechanisms and antimicrobials, making the infection extremely difficult to treat.^80^

A number of infections involving bones and joints are biofilm infections.^83^ A biofilm is a polysaccharide-based extracellular matrix surrounding the multistructured, diversified colony of immobilized microorganisms.^84^ These microorganisms are in the stationary growth phase, are metabolically less active, and can endure host mechanisms and most antimicrobials, therefore making biofilm infections difficult to treat.^83^ The most common biofilm-forming species include *S. aureus*, *S. epidermidis*, group A *streptococci*, and *Pseudomonas aeruginosa*.^84^ The three most common orthopedic biofilm infections include chronic osteomyelitis, periprosthetic joint infections, and implant-associated osteomyelitis of long bones.^83^

**Figure 2:**
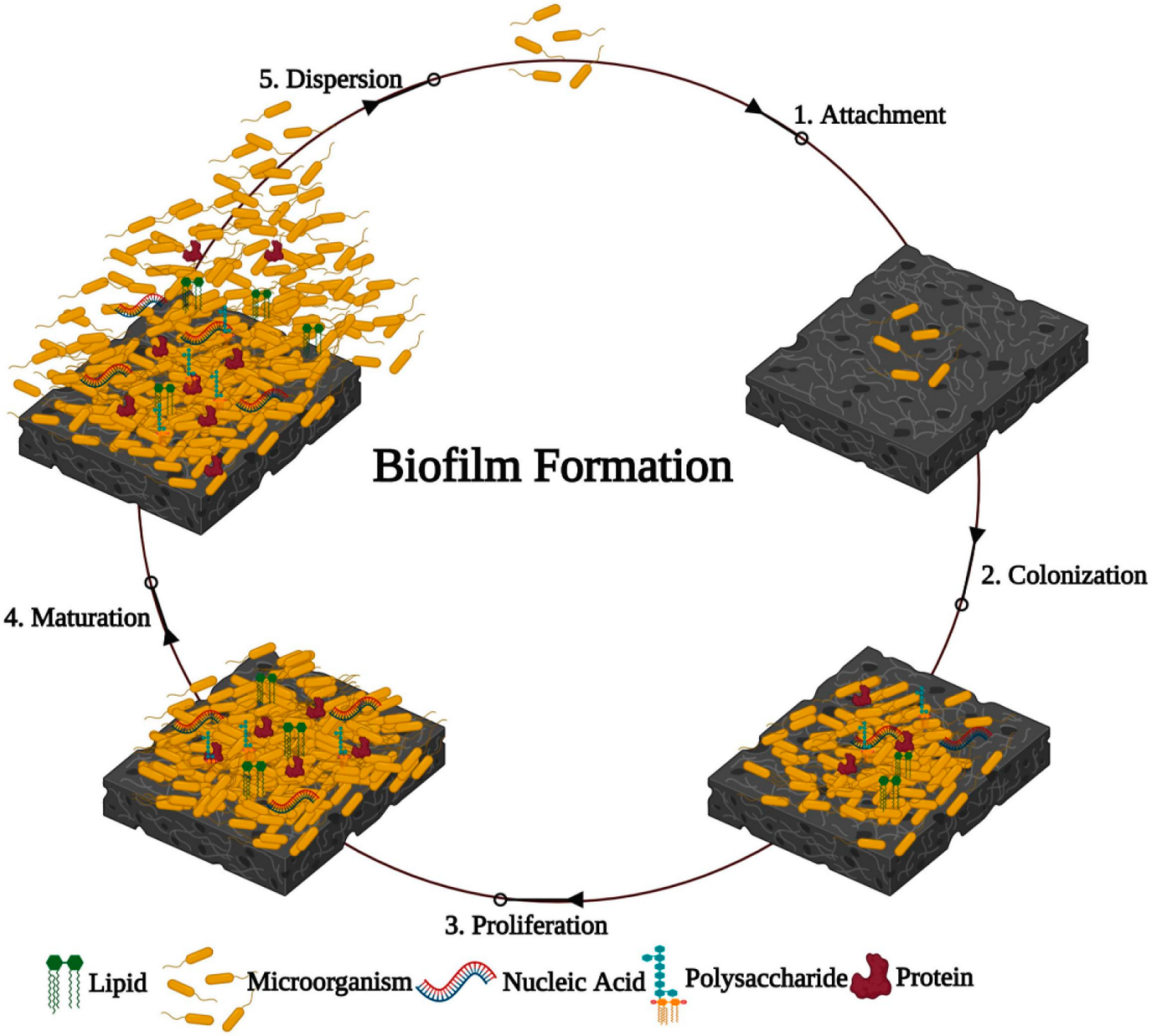
Stages involved in biofilm formation. For example, *S. aureus* cells, which are the most common BJI pathogen, could attach to abiotic and biotic surfaces via hydrophobic interactions and microbial surface adhesive matrix molecules, respectively. After attachment, it develops into a mat-like structure of cells composed of extracellular DNA and a proteinaceous matrix. Then, cells are released from the biofilm via nuclease-mediated extracellular DNA degradation to allow for the formation of three-dimensional microcolonies. There is also a rapid cell division for maturation. Finally, the dispersal of cells takes place via protease activation^86^ [Reprinted with permission from ref (865). Copyright 2021 Frontiers].

Microorganisms follow a similar process in bacterial biofilm formation: mainly, initiation on which the biofilm grows, microcolony formation where both pathogen and nonpathogen microbes infiltrate the biofilm, and maturation along with extracellular polymeric substance production in which natural polymers (polynucleotides, polypeptides, and polysaccharides) of high molecular weight (>0.5–2 × 10^6^ Da) are secreted by microorganisms into their environment and provide adhesion.^85^ Then, finally, individual cells disperse as depicted in Figure 2.

The increased resistance of biofilms to antibiotics allows biofilm-based infections to persist despite antibiotic therapy. They can withstand and survive harsh environmental conditions and even high-dose antimicrobial agents.^87^ To treat pathogenic biofilms from a patient, typically, 10–1000 times higher doses are required than an identical strain in its singular form.^87^ This is due to the embedded bacterial cells getting an optimal defense mechanism against the mechanism of action of antibiotics and the immune system of the host. Some of the reasons include an altered gene expression in biofilm-specific resistance genes (e.g., efflux pumps or exclusion of antibiotics), less sensitivity of antibiotics against the slower growth rate, and reduced metabolic activity of cells. Furthermore, there can be degradation of antibiotics by enzymes in the biofilm matrix, impaired penetration of antibiotics into the biofilm matrix, a stress response to hostile environmental conditions (e.g., leading to an overexpression of antimicrobial agent-destroying enzymes), and an altered environment inside the biofilm matrix (pH, oxygen content).^88−90^

Despite biofilm matrices not inhibiting drug diffusion completely, the payload is required to bind to the components of the matrix or the bacterial membranes. Restricted penetration of antimicrobials may occur as negatively charged polysaccharides restrict permeation of positively charged payload.^88^ The scarcity of nutrients and oxygen in biofilm is another underlying cause of biofilm-associated antimicrobial resistance.

Bacterial biofilms are also composed of persister cells, which are neither growing nor dying when they are exposed to antimicrobials, consequently leading to MDR.^91^ Persister cells are metabolically inactive, subset of dormant, phenotypic regular bacteria with a high tolerance to antibiotics without undergoing any genetic change. They form in response to several environmental factors, such as nutrients and oxygen deprivation, oxidative stress, and DNA damage. Persister cells are specialized survivors which are distinct from both growing and stationary cells, and they are the only cells to survive treatment with high doses of antimicrobials.

Management of biofilm-forming pathogens is difficult to treat even with high doses of antibiotics and can ultimately lead to sepsis and, if left untreated, can result in death.^91^ Hence, new and effective approaches are urgently needed.

## 4 Nanodrug Delivery Systems as a “Silver Bullet” to Tackle AMR in BJI

There has been an increase in the development and optimization of novel drug delivery systems capable of altering the performance of conventional drug delivery systems in recent years. Among these novel drug delivery systems are nanodrug delivery systems (NDDSs), which are based on nanometric sizes. NDDSs have been shown to improve some of the drawbacks of traditional drug delivery systems, such as low bioavailability, off-target drug delivery, and frequent and inconvenient dosing.^92,93^ In this paper, NDDSs refer to any materials that possess a nanometric dimension, i.e., <1000 nm,^94^ and are used as a carrier to deliver API successfully to circumvent or improve outcomes in the treatment or management of AMR in BJI.

**Figure 3:**
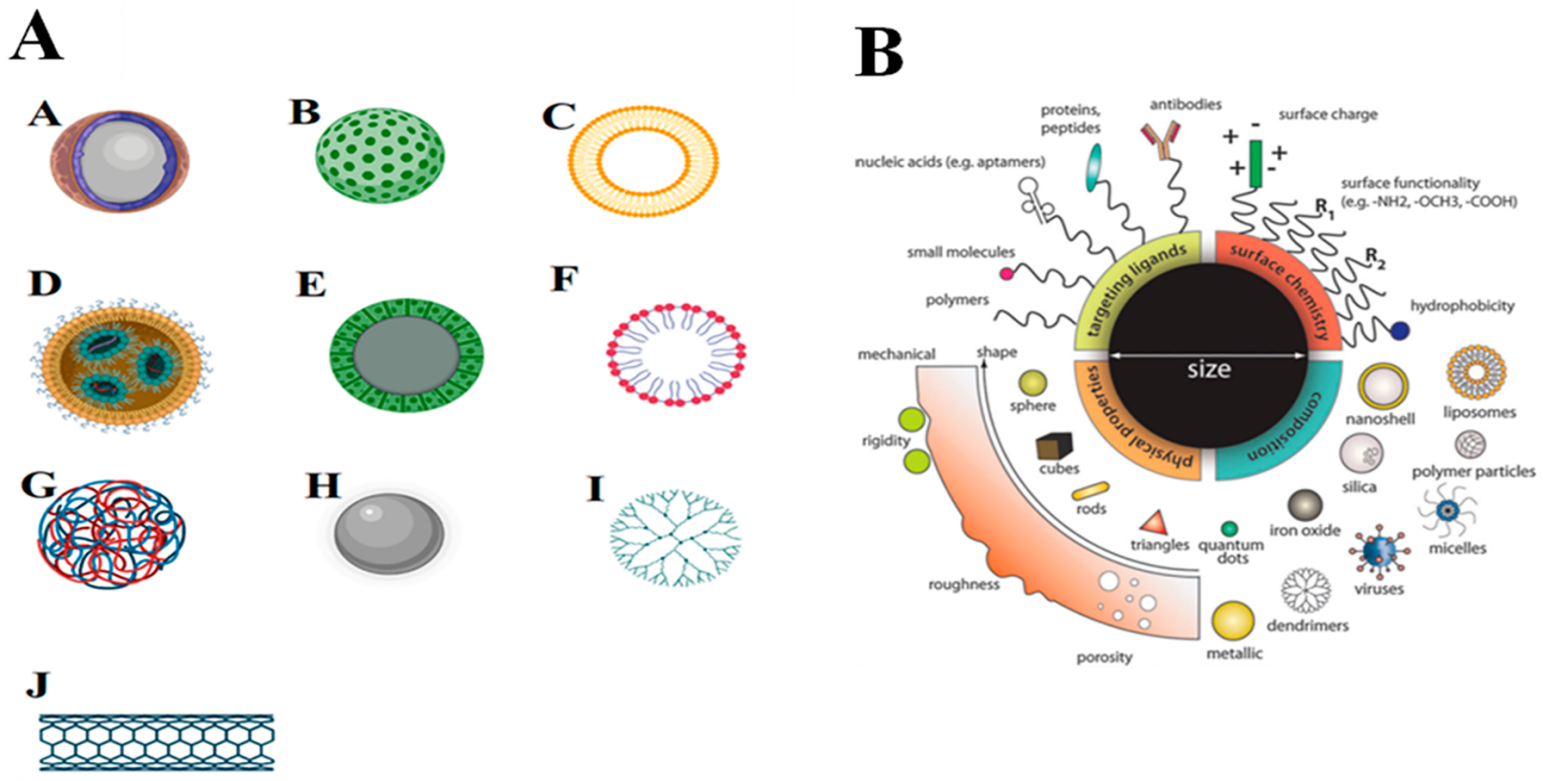
Summary of the different types of NDDSs used to deliver APIs (A; A: nanospheres; B: mesoporous silica nanoparticles; C: liposomes; D: solid lipid nanoparticles; E: solid core mesoporous nanoparticles; F: micelles; G: polymeric nanoparticles; H: silver nanoparticles; I: dendrimers; J: carbon nanotubes) and NDDS modularity allowing improved performance (B) [Reprinted with permission from ref (95). Copyright 2011 Royal Society of Chemistry].

There are many types of NDDSs that are generally characterized by fundamental components. Theoretically, the fact that most NDDSs can be modulated offers an infinite medium of nanomaterials possessing different properties, making NDDSs loaded with therapeutics more versatile than either small molecules or larger micron-sized particles in performing complex functions within AMR treatment. The types of nanomaterials and their modulatory aspects are depicted in Figure 3 with a summary of the advantages and disadvantages of nanoantibiotics provided in Table 2.

**Table 2:** Summary of the Advantages and Disadvantages of Nanoantibiotics^96^

advantages	disadvantages
Good biocompatibility	Nonbiodegradable inorganic and magnetic NPs may exhibit undesirable properties in humans
Low toxicity	Translation from lab to clinical use is still a challenge
Easy functionalization	
Variety of encapsulation strategies	

While the use of nanomaterials to treat BJIs^97^ and to treat infections such as MRSA have been reported,^98−102^ the use of NDDSs to treat microbes that are resistant to commonly used antibiotics specifically for BJIs is not widely reported. However, a handful of studies have shown the ability to use NDDSs to circumvent AMR in BJIs.

### 4.1 Lipid-Based Nanodrug Delivery Systems

Lipid-based nanodrug delivery systems such as liposomes, niosomes, and solid lipid nanoparticles have long been investigated in the treatment of various infectious diseases. They are arguably the original templates upon which nanodrug delivery was developed. Unsurprisingly, they have been used in the quest to circumvent AMR and specifically in BJIs with relative success.

Li et al. investigated the use of a combination of daptomycin (DAP) and clarithromycin (CLA) against MRSA infections. The coencapsulated novel liposomal formulation was developed at an optimal drug ratio.^103^ Both *in vitro* and *in vivo*, the novel coloaded liposomal formulation demonstrated more effective antibacterial activity than individual drug-containing liposomes and significantly prolonged the survival rate of infected mice. Furthermore, coloading CLA significantly reduced the amount of DAP required without sacrificing clinical efficacy, potentially lowering the risk of potential toxicity.^103^ The findings of this study show great promise for the further development of an alternative DAP-based approach to the treatment of severe infections, with increased therapeutic efficacy and safety.

Using the aforementioned study findings, nanoencapsulated DAP was developed and observed to have enhanced sterilization of the infectious sites after 4 and 14 days of treatment, while daily systemic daptomycin treatment for 4 days was ineffective.^104^ In these experiments, a rabbit osteomyelitis model was used and an evaluation of the activity of a gel loaded with DAP encapsulated in lipid nanocapsules (LNC-DAP) was compared to that of free intravenous (i.v.) DAP.^104^ A MRSA strain isolated from a blood culture belonging to sequence type 8 [ST8] and clonal complex 1 (CC1) with a minimum inhibitory concentration (MIC) of 0.5 μg/mL for free DAP and LNC-DAP was utilized. The percentage of negative cultures was >75% after 96 h following a single local administration of LNC-DAP with no negative cultures observed with the i.v. DAP regimen.^104^

Onyeji et al. explored the use of lipid-based NDDS to circumvent MRSA.^105^ The antibacterial effects of liposomal vancomycin (VNC) and teicoplanin (TPN) against intracellular MRSA were evaluated using a macrophage infection model. Monocytes derived from human blood were cultured for 7 days to obtain adherent macrophages. The data demonstrated that the uptake of each drug by macrophages was markedly enhanced by liposomal encapsulation. Following phagocytosis and removal of residual extracellular MRSA, the infected macrophages were exposed to clinically achievable concentrations of teicoplanin and vancomycin.^105^

**Figure 4:**
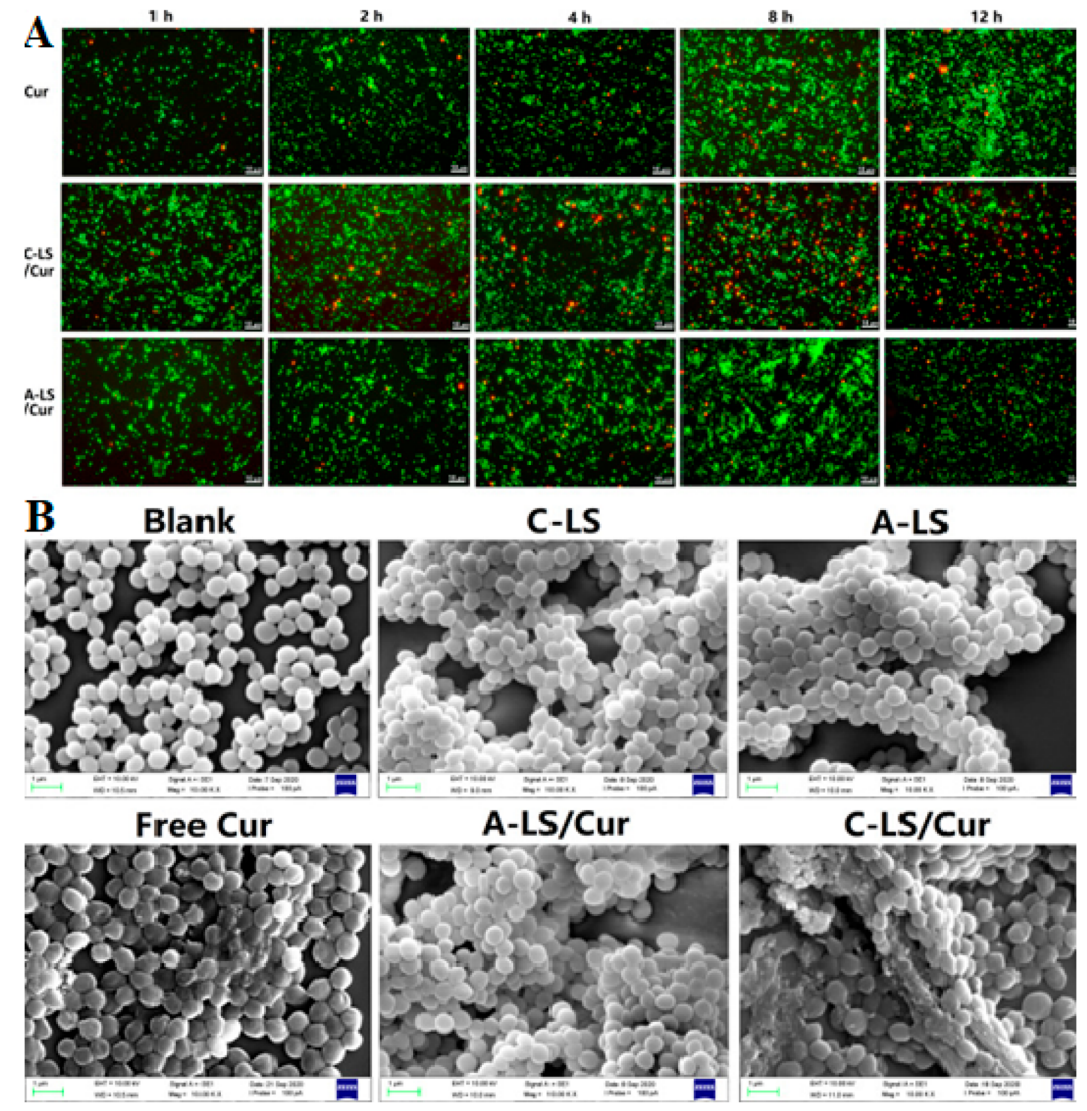
Images of *S. aureus* BHYB3-2 stained by a live/dead kit after incubation with free Cur, C-LS/Cur, and A-LS/Cur at 37 °C for 1, 2, 4, 8, and 12 h (A). SEM micrographs of *S. aureus* BHYB3-2 treated for 12 h with negative control, C-LS, A-LS, free Cur, A-LS/Cur, and C-LS/Cur (B) [Reprinted with permission from ref (106). Copyright 2021 Elsevier B.V.].

Guo et al. developed cationic liposomal curcumin (C-LS/Cur), and their effect against antibiotic-resistant *S. aureus* was assessed.^106^ It was observed that the cationic liposomes loaded with Cur had superior activity against *S. aureus*. This was attributed to the negatively charged *S. aureus* favoring electrostatic interactions rather than intercalation with cationic liposomal vesicles at the beginning of the endocytic process, thereby effectively delivering the payload to its targets.^106^ In an attempt to give validity to the hypothesis, the investigators monitored zeta potential variation and collected visual evidence through fluorescence confocal microscopy (FCM) and transmission electron microscopy (TEM) as depicted in Figure 4.^106^

In addition to these characterizations, confocal laser scanning microscopy (CLSM) and binding kinetics were determined using biolayer interferometry (BLI). Moreover, an excellent therapeutic efficacy of the cationic liposome technology against invasive murine infection was also observed, which was due to the enhanced accumulation and retention in the targets.^106^

Ayre and co-workers combined gentamycin (GEN) loaded liposome technology with the use of poly(methyl methacrylate) (PMMA) cement for bone use.^107^ The novel combination resulted in a controlled and gradual release of payload over a 30-day period, as well as increased toughness, bending strength, and Vickers hardness of the cement without a change in polymerization or molecular structure. This novel combined technology has the potential to significantly reduce infections in cemented joint replacements, potentially leading to improved patient quality of life and lowering costs of healthcare.^107^

### 4.2 Inorganic Nanodrug Delivery Systems

To overcome challenges with conventional drug delivery systems, inorganic materials such as metals and semiconducting materials such as silicon have long been used in nanodrug delivery. Their application in the treatment of microbial infections has been investigated.^108,109^ There has been a concerted effort to use this technology to treat BJIs that are resistant to traditional therapy.

Jiang and co-workers explored the use of nanohydroxyapatite (nHA) pellets as carriers for VNC in the treatment of chronic osteomyelitis and bone defects due to MRSA strains.^110^ Following an initial rapid release into circulation, the payload concentration remained effective in soft tissue and osseous for 84 days after debridement. Within three months, all rabbits in the experimental group recovered from osteomyelitis without infection recurrence, and the bone defects were partially repaired, whereas infection and bone defects persisted in the control and blank groups.^110^

Nanomaterials made from metals have found significant use in antimicrobial therapy. The use of silver-based (Ag) species on orthopedic implants to prevent implant-associated infection is gaining popularity. AgNP-coated proximal femur or tibia prostheses, external fixation pins, and AgNP-loaded bone cement were engineered for surgical orthopedic interventions.^111^ Despite their many successes, not many metal-based nanomaterials have been developed and demonstrated efficacy against resistant strains of bacteria that cause BJIs.

Notwithstanding, Alt and colleagues synthesized nanoparticles made from silver (AgNP), tagged them with GEN, and investigated their use in MRSA.^112^ These were investigated by loading the AgNPs/GEN in poly(methyl methacrylate) (PMMA) cement, which is used as indispensable components for anchoring joint replacement prostheses in the bone.^112^ Unloaded and PMMA cement loaded with 2% GEN did not exhibit any antibacterial activity against MRSA and methicillin-resistant *Staphylococcus epidermis* (MRSE). However, the cement loaded with 1% AgNP completely inhibited the proliferation of MRSA and MRSE.^112^ The novel AgNP bone cement did not show any significant differences compared to the nontoxic control group with regard to safety and toxicity.^112^

Qin et al. successfully developed a silver nanoparticle (AgNP) system that was capable of reducing biofilm on titanium implants.^113^ They achieved this by using silver plasma immersion ion implantation (PIII) in which the Ag NPs are manufactured *in situ* and immobilized on titanium. The antibiofilm activity of immobilized AgNPs was evaluated *in vitro* and *in vivo* using a biofilm-producing strain, *S. epidermidis*.^113^ Furthermore, the technology demonstrated a reduction in biofilm formation *in vitro* by inhibiting bacterial adhesion and icaAD transcription. *In vitro*, immobilized AgNPs provided effective defense against multiple cycles of bacterial attack, demonstrating a Ag release independent mechanism. Further assessments via microbiological cultures, radiography, and histopathology reviewed that the functionalized surface has the ability to reduce the risk of implant-associated periprosthetic infection.^113^

Silk fibroin nanoparticles (SFNPs) have also been explored for treating severe bone infections in a rat tibia osteomyelitis model.^114^ The SFNPs were used to deliver VNC to infection sites, and the activity was compared against the free drug. Furthermore, the SFNPs were loaded in silk scaffolds with these technologies being assessed for drug release at two pH values, viz., 4.5 and 7.4. The results of the *in vitro* drug release from the SFNPs and SFNP-loaded silk scaffolds showed favorable drug release at both pH values. *In vivo* assessments were then performed by injecting 8 × 10^6^ CFU MRSA in the tibia of rats to induce severe osteomyelitis disease.^114^ Radiographic and histopathological analyses were performed to evaluate the effectiveness of treatment after 42 days. VNC-loaded SFNP entrapped in scaffolds reduced bone infections at the defect site more effectively than any other treatment group. The authors concluded that this novel delivery system, which demonstrated desirable biocompatibility and sustained release properties, should be investigated further in the context of osteomyelitis treatment.^114^

Nie and co-workers explored the efficacy of D_6_ and UBI_29–41_ peptides in targeting sites of bone infection.^115^ The researchers explored the fabrication of bone-and-bacteria dual-targeted mesoporous silica NPs with D_6_ and UBI_29–41_ peptides to target bone infection sites. The novel mesoporous silica NPs loaded with VNC were able to control release in bone infection sites. The data obtained showed that the dual-targeted mesoporous silica NPs have excellent bone and bacteria targeting efficacy, excellent biocompatibility, and effective antibacterial properties *in vitro*.^115^ Moreover, in an *in vivo* rat model with MRSA bone infection, bacteria growth was significantly inhibited in the absence of cytotoxicity, allowing for the early treatment of implant-related infection.^115^

Jia et al. developed two technologies to treat osteomyelitis caused by MRSA.^116^ The two groups of implants were composed of 10% w/w teicoplanin (TEC)-loaded borate bioactive glass (TBG) or calcium sulfate (TCS). Among the critical quality attributes assessed in these experiments were the assessment of the technology to release TEC *in vitro* and to cure MRSA-induced osteomyelitis in a rabbit model.^116^ In the *in vitro* model using phosphate-buffered saline (PBS), both groups of implants exhibited a sustained release of TEC at a therapeutic level for up to 21 to 28 days.^116^ In the *in vivo* rabbit model, infected tibiae were treated by debridement. This was followed by stratification into groups that received implantation differently, viz., TBG or TCS pellets or i.v. injection with TEC, or were left untreated. Data from the evaluation, which was carried out 6 weeks after implantation, showed that animals implanted with TBG or TCS pellets had radiological and histological scores, rates of MRSA-positive cultures, and bacterial loads that were significantly lower than those of animals treated intravenously or preoperatively.^116^ Furthermore, the level of bone regeneration was higher in the defects treated with the TBG pellets. The novel technology developed demonstrated that local TEC delivery was more effective than i.v. administration for the treatment of MRSA-induced osteomyelitis.^116^

Krishnan et al. investigated the efficacy of a nanocomposite fibrous scaffold composed of silica-coated nanohydroxyapatite-gelatin reinforced with poly-l-lactic acid yarns and loaded with VNC for the treatment of MRSA-induced osteomyelitis in rat models.^117^ The payload was incorporated either during scaffold synthesis (SE-V) or loaded directly after the development of the scaffold (SA-V) at 5%w/w and 15%w/w. It was observed that the payload release was sustained over a 30-day period and demonstrated antibacterial activity against MRSA.^117^ Moreover, implanting the composite scaffold into an osteomyelitic rat femur resulted in a significant bacterial reduction, more markedly with 15%w/w drug loading, and its efficacy was comparable to that of a commercial graft. It was further observed that both entrapped and absorbed payload composite scaffolds promoted bone regeneration in 90 days, with no distinguishable difference between them. However, the commercial graft resorbed faster and had bone voids at the defect site after 3 months. The data obtained from these studies suggested that the nanocomposite fibrous scaffold containing VNC could be proposed as a bifunctional graft capable of reducing bacterial infection, while subsequently engineering new bone in osteomyelitic patients.^117^

Cao et al. developed a novel VNC-loaded bone-like hydroxyapatite/poly(amino acid) (V-BHA/PAA) scaffold assessing its osteogenic^118^ and antibacterial activity.^119^*S. aureus* and MRSA were both significantly and consistently eradicated by V-BHA/PAA in both *in vitro* and *in vivo* testing. Additionally, the bactericidal effect of the novel technology outperformed that of commonly used poly(methyl methacrylate) loaded with VNC (V-PMMA). *In vitro*, the antibacterial effects of V-BHA/PAA on *S. aureus* and MRSA lasted longer than 4 weeks while those of V-PMMA lasted only 2 weeks. In the chronic osteomyelitis model, the curative rate for V-BHA/PAA was 75% for regular *S. aureus* and 67% for MRSA infection, which was significantly higher than V-PMMA rates of 50% and 42%, respectively.^119^

Al Thaher and co-workers manufactured chlorhexidine, a widely used topical antibiotic compound, coated silica nanoparticles with prolonged drug release capabilities, and incorporated them into commercial formulation PMMA bone cement without a significant reduction in mechanical performance.^120^ Furthermore, the silica-NP loaded PMMA bone cement displayed enhanced antimicrobial activity against different bacterial species encountered in prosthetic joint infections, which included clinical isolates with previously demonstrated resistance to GEN. Moreover, the cytocompatibility assessments also demonstrated the non-inferior performance of the bone cement containing chlorhexidine-coated silica-NP to the commercial product. While this study was not performed in animal models, it formed an important basis for further assessments due to the utilization of commercially available PMMA bone cement that is commonly already in use.^120^

It is important to note that, generally, the use of inorganic nanodrug delivery systems is associated with more toxicity compared to other nanodelivery systems such as those based on lipids or polymers, and this presents a significant limitation that could result in lesser use.

### 4.3 Polymer-Based Drug Delivery Systems

Polymeric nanomaterials have long been used to overcome several of the drawbacks associated with conventional drug delivery. Polymers used in drug delivery have provided improved outcomes in many disease states including infectious diseases.^121−123^

Guo and colleagues developed a lipid–polymer hybrid NP designed to load the antibiotic linezolid (LIN) with a specific focus on evaluating the potential for this novel nanoantibiotic to achieve significant *in vitro* activity against these intracellular and biofilm-embedded MRSAs.^124^ The optimized LIN-loaded lipid-polymer NP (LIN-LNP) formulation showed high entrapment efficiency and controlled release characteristics over 5 days. Despite it achieving lower activities against USA300-0114, CDC-587, and RP-62A in planktonic form, LIN-LNP exhibited substantial superiority against the intracellular MRSA reservoir of osteoblast cells. The LIN-LNP exhibited significant differences of 87.0-fold, 12.3-fold, and 12.6-fold in intracellular activities when compared to the free drug in CFU/mL at 2, 4, and 8 μg/mL linezolid concentrations, respectively.^124^ Moreover, LIN-LNP also exhibited 35–60% suppression of MRSA biofilm growth when compared to the free drug. These enhanced intracellular and antibiofilm activities were attributed to the extensive accumulation of the technology inside the MRSA-infected osteoblasts and biofilms as revealed in the confocal microscope images.^124^

A research team headed by Almaaytah designed and developed a novel potent ultrashort antimicrobial peptide (AMP) known as RBRBR and entrapped it in chitosan nanoparticles (RBRBR-CS-NP) utilizing an anionotropic gelation technique.^125^ The encapsulated peptide demonstrated accumulative sustained release for 2 weeks. Moreover, a significant decrease (1000-fold) in *S. aureus* counts with 98% inhibition of biofilm formation was observed with no demonstrable toxicity against mammalian cells and human erythrocytes.^125^

To codeliver lidocaine, VNC, and ceftazidime, Hsu et al. developed a novel electrosprayed multi-API-loaded NP system utilizing poly(d,l-lactide-co-glycolide) (PLGA) as the polymer sheath. The technology was intended for intra-articular injection for the treatment of local septic arthritis.^126^ The biodegradable electrosprayed technology released high concentrations of payload into the synovial knee tissue of rabbits for more than 14 days which was well above the MIC_90_ for *S. aureus*.^126^

### 4.4 Stimuli-Responsive Nanomaterials

Stimuli-responsive drug delivery in the treatment of bacterial infections is an attractive technique of targeted drug delivery making use of specific infection-related stimuli to improve treatment outcomes while avoiding the normal physiological conditions reducing off-target delivery.

Utilizing this, Pornpattananangkul et al. fabricated phospholipid liposomes that were stabilized by gold nanoparticles (AuChi). The novel technology payload release was responsive to bacterial toxin.^127^ The AuNPs were previously functionalized with chitosan and adsorbed to the surface of the liposomes to provide stability to the liposomes and prevent unintended antibiotic leakage. The AuChi liposomes are effectively released as the model payload in response to the toxin secreted by *S. aureus*.^127^

Albayaty and co-workers designed a novel enzyme-sensitive copolymer micelle that is susceptible to cleavage by lipases/esterases produced by bacteria, including *S. aureus* and *P. aeruginosa* that resulted in the successful targeted release of chlorhexidine in bacterial biofilms.^128^ This technology not only resulted in superior payload permeability (∼71%) but also resulted in a >60% maximum reduction in biofilm biomass.^128^

Heat as a stimulus in the inactivation of biofilms has the potential to be very useful. With this in mind, hyperthermia using super paramagnetic iron oxide nanoparticles (SPIONs) can be expected to play a critical role in the inactivation of bacterial biofilms.^129^ Park et al. developed SPIONs for this purpose and based on the heating experiments in a water bath were able to inactivate the bacterial biofilm. Using this method, it was possible to preferentially heat specific areas, and the heating temperature was easily manipulable by controlling the concentration of the SPION solution, magnetic field intensity, and heating time.^129^ Furthermore, this technology does not require any toxic chemicals such as chlorine while also being recyclable. The technology was utilized in the removal of biofilms from various medical devices. It has the potential to be utilized for therapeutic applications and combined with affinity and targeting techniques such as antigen–antibody or enzyme–substrate binding for more accurate control of the bacterial biofilm removal.^129^

Similarly, it was demonstrated that an injectable, thermoresponsive, hyaluronic acid-based hydrogel loaded with GEN and VNC outperformed current clinical practice on the treatment chronic MRSA orthopedic device-related infections in a sheep model.^130^ This study was designed and developed to further the findings of the applicability of local application of a GEN-loaded biodegradable thermoresponsive poly(*N*-isopropylacrylamide)grafted hyaluronic acid (HApN) hydrogel, which allowed for fracture healing following clearance of a high *S. aureus* load in a rabbit model.^131^

Li and colleagues tailored a nanosystem based on l-lysine carbon dots (CDLys) and modified it by use of pH-responsive copolymer with the intended application as microenvironment-responsive antibiofilm agents.^132^ The self-assembled nanostructure was capable of rapidly diffusing in the mature *S. aureus* biofilm and effectively responding to the acidic microenvironment of the biofilm. Once there, the nanostructure could be triggered to disassemble into two parts, viz., −NH_2_ ended copolymer and CDLys.^132^ The copolymer has the ability to target negatively charged bacteria surfaces and showed enhanced antibacterial ability for the protonated −NH_2_ groups.^132^ On the other end, the released CDLys diffuse throughout the dense biofilm and generate substantial amounts of reactive oxygen species (ROS) for bacterial death. It is also worth noting that the PEGylation of the nanostructure results in a technology that was nonhemolytic and demonstrated excellent biocompatibility to fibroblast cells.^132^

Chen et al. developed a novel system promoting the on-demand release of antimicrobial peptides (AMPs) in and around the affected joint area and implant when bacterial infection occurs and lowers the surrounding pH.^133^ It was termed a Pandora’s box approach. This technology was loaded with HHC36 peptide inside specially designed titanium nanotubes (Ti-NTs) enclosed via surface modification with a pH-responsive molecular gate. The PMAA swells under physiological pH conditions but collapses under the acidic pH conditions that occur under bacterial infection, allowing the release of AMPs.^133^ This novel technology exhibited excellent activity against MRSA, *E. coli*, and *P. aeruginosa*, thus representing a novel stimuli-responsive drug delivery technology against drug-resistant BJIs.^133^

Hu and co-workers designed a 14 nm surface-adaptive mixed charged zwitterionic gold nanoparticle (AuNP-N-C), which was fabricated with mixed self-assembled monolayers (SAMs) consisting of strong electrolytic (10-mercaptodecyl) trimethylammonium bromide (HS-C_10_-N_4_) and weak electrolytic 11-mercaptoundecanoic acid (HS-C_10_-COOH).^134^ The surface-adaptive mixed charged zwitterionic AuNP-N-C was capable of effectively adhering to bacteria and aggregating rapidly to the acidic biofilm. However, the AuNP-N-C was also capable of forming a stable dispersion in healthy tissues. The aggregated AuNP-N-C with enhanced near-infrared (NIR) absorbance could effectively convert NIR light energy into localized heat, resulting in thermal ablation of the MRSA biofilm. Simultaneously, the dispersive AuNP-N-C exhibited no damage to healthy tissues.^134^ The novel API free pH-responsive gold nanoparticles that were manufactured have great potential in the treatment of bacterial infection, including drug-resistant bacteria and their biofilms.^134^

Iron oxide NPs (FeNPs) have been widely used in biomedical research as a consequence of their unique properties, good biocompatibility, low cytotoxicity, and simple synthesis.^135^ Li et al. demonstrated the use of magnetic FeNPs as a modular tool for disrupting biofilms and targeting the infection site as a possibility. The optimization of the technology involved modulating the NPs with regard to the size and shape of the NPs. NPs with sizes of 8, 11, and 70 nm were manufactured and used as a physical means to target biofilms with a magnetic field resulting in controlled delivery, which is not a possibility with conventional antibiotic therapy.^135^ In an attempt to isolate the physical effect of the magnetic NPs (MNPs) from any potential chemical interactions, silica was used to coat the MNPs. The date generated demonstrated that, despite all MNP sizes being capable of removing biofilms, the application of a magnetic field improved the effects.^135^ Furthermore, the use of hyperthermia with a high frequency alternating current (AC) field resulted in the 70 nm MNPs having the least effect among the three sizes of NPs as a result of the temperature of 70 nm particle solution not increasing as those of the 8 and 11 nm MNP solutions. However, the 70 nm MNPs presented a strong magnetic response and offered good antibiofilm performance in the direct current (DC) rotating magnetic field. The 8 and 11 nm particles showed a similar antibiofilm effect.^135^

Hu et al. developed a synergistic antibiofilm system that targeted the biofilm microenvironment and was designed using the multifunctional nitric oxide (NO) donor as a generalist to improve the photodynamic therapy (PDT) efficacy.^136^ The supramolecular α-CD-Ce6-NO-DA nanocarriers were fabricated via the host–guest interactions between α-CD-based pro-drugs (α-CD-NO and α-CD-Ce6) and pH-sensitive copolymer PEG-(KLAKLAK)_2_-DA.^136^ The negatively charged surface of the synthesized -CD-Ce6-NO-DA became positively charged at the acidic biofilm pH, allowing for excellent penetration and accumulation into the biofilm, which is required for effective bacteria killing in the biofilm. In the physiological environment, the -CD-Ce6-NO-DA was relatively stable, with little NO release. The technology demonstrated GSH-triggered fast NO release when penetrating biofilm with overexpressed glutathione (GSH).^136^ The GSH-triggered NO release behavior not only generated massive NO with high bactericidal activity but also reduced the GSH concentration in the biofilm, which was beneficial to improving PDT efficiency. Furthermore, via the reaction between NO and reactive oxygen species (ROS), peroxynitrite anions (ONOO) with higher toxicity to bacteria were formed, improving the PDT efficiency even further. As a consequence of the low photosensitizer dose and laser intensity, the technology had little effect on healthy tissues.^136^

Ideally, an implanted material for the purposes of bone treatment should possess dual functionality with regard to antibacterial therapy and bone tissue regeneration. Ding and co-workers envisaged and fabricated an enzyme-responsive nanoplatform to treat implant-associated bacterial infection and accelerate tissue regeneration *in vivo*.^137^ AgNPs were first pre-encapsulated in mesoporous silica nanoparticles using a one-pot method. LBL@MSN-AgNPs were created by sequentially assembling poly-l-glutamic acid (PG) and poly(allylamine hydrochloride) (PAH) using the layer-by-layer (LBL) assembly technique. Subsequently, the LBL@MSN-AgNPs were deposited on the surface of polydopamine-modified titanium (Ti) substrates. Ti substrates modified with an LBL@MSN-Ag nanocoating had an excellent antibacterial effect *in vitro*. The modified implants were successful in treating the bacterial infection *in vivo* in a rat model with a femur defect that had been infected with bacteria. In addition, the results of the hematoxylin-eosin staining, micro-CT, and Masson’s trichrome staining demonstrated that the modified implants significantly accelerated osteogenesis for 4 weeks after implantation.

In recent times, a considerable amount of attention has been drawn to bacteria-triggered, self-defensive antibacterial coatings (BT-SDACs) as they have demonstrated significant clinical potential as a consequence of their ability to release antimicrobial agents rapidly and locally on-demand in response to endogenous stimuli surrounding infection sites on the surface of biomedical implants.^138−140^

**Figure 5:**
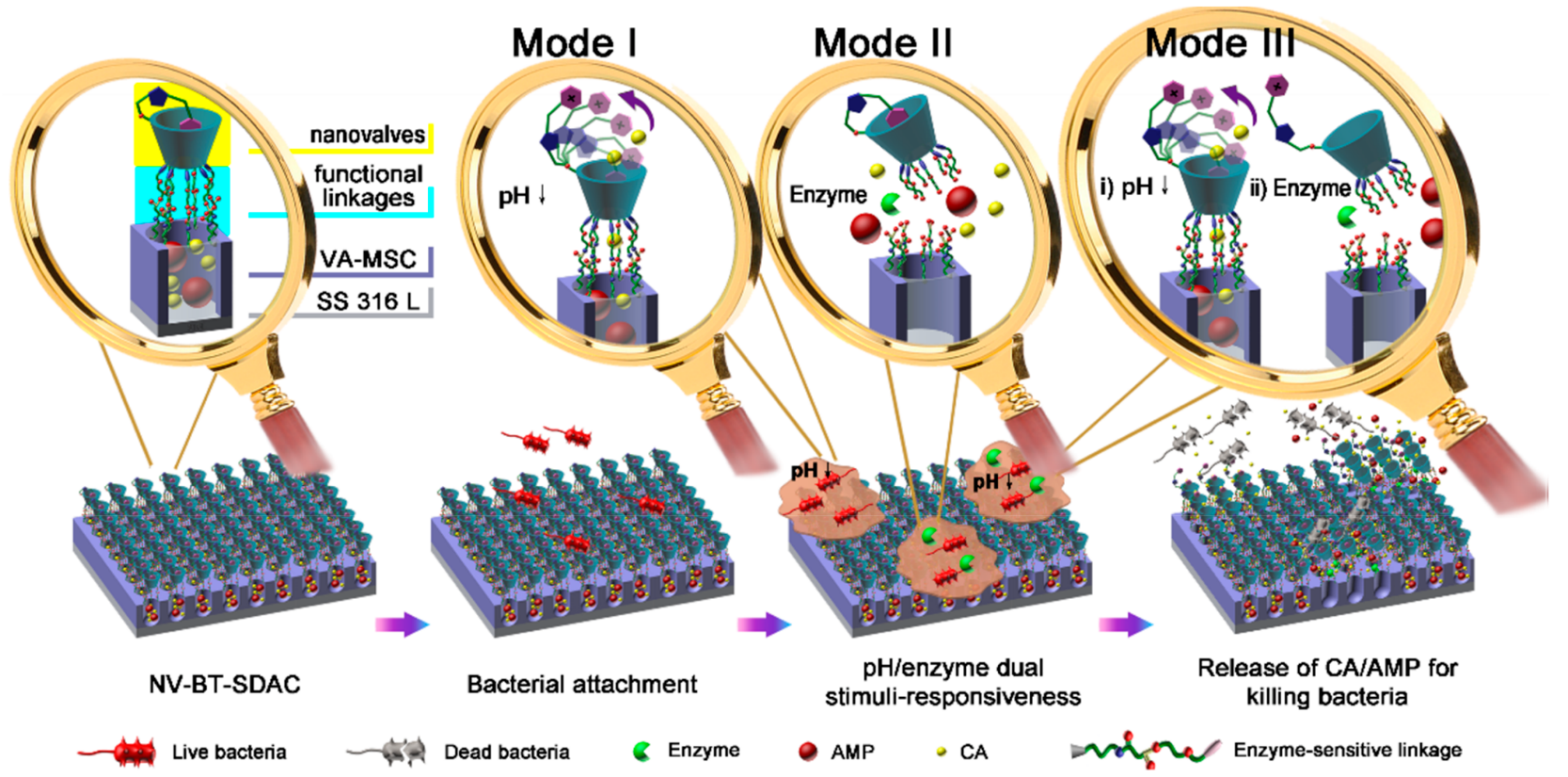
Schematic representation of the structure and working mechanisms for NV-BT-SDAC deposited on a metallic base [Reprinted from (141). Copyright 2017 American Chemical Society].

Wang et al.^141^ demonstrated that the use of a novel nanovalve-based, bacteria-triggered, and self-defensive antibacterial coating (NV-BTSDAC) loaded with cinnamaldehyde (CA) and ampicillin (AMP) had the potential to eradicate *S. aureus*, *E. coli*, or MRSA from implants. Three different release modes, depicted in Figure 5, were obtained after applying pH/enzyme stimuli, which frequently appear in local infection sites, viz., CA release triggered by pH via reversible structural transformation of nanovalves; enzyme-induced corelease of CA and AMP as a result of functional linkage cleavage, and ordered action of pH and enzyme stimuli resulting in the sequential release of CA and AMP. When tested against *S. aureus*, *E. coli*, and MRSA, NV-BT-SDAC demonstrated excellent antibacterial and antiadherent properties. The pH/enzyme dual-stimuli responsiveness improved response sensitivity, and the synergistic interaction of CA and AMP demonstrated acceptable antibacterial activity against antibiotic-resistant bacteria.

A summary of the nanomaterials against bacteria associated with BJIs is provided in Table 3.

**Table 3:** Summary of Nanosystems Utilized in BJIs

type of nanosystem	nanocarrier	API	composition	CQA	model	significant findings	ref
Lipidic Nanodrug Delivery System	Liposomes	Clarithromycin and Daptomycin	Hydrogenated soy phosphatidylcholine (HSPC)	PS: 98.2 ± 2.51 nm	*in vitro* and *in vivo*	Daptomycin and clarithromycin co-delivery	(103)
			methylpolyethyleneglycol-1,2-distearyl-phosphatidylethanolamine conjugate (mPEG_2000_-DSPE)	PDI: 0.251		significant anti-MRSA activity	
				EE: CLA 92.94% ± 1.21%; DAP 94.71% ± 1.37%		lower concentration of API required	
	LNC in gel	Daptomycin		PS: 78.8 nm	Rabbit osteomyelitis model	Enhanced sterilization up to 14 days post-treatment	(104)
				PDI: 0.15			
	Liposomes	Vancomycin and Teicoplanin	egg phosphatidylcholine		*In vitro* macrophage model	A marked increase in teicoplanin or vancomycin uptake into macrophages	(105)
			diacetylphosphate				
			cholesterol				
	Liposomes	Curcumin	Dioleoyl-3-trimethylammonium propane (DOTAP)	PS: 142.34 ± 2.57 nm; ZP: +15.91 ± 1.56 mV	Mouse model	Improved biocompatibility of CUR	(106)
			Dipalmitoylphosphatidylcholine (DPPC)	PDI: 0.241 ± 0.037		Improved pharmacokinetics of CUR	
			Cholesterol	DL: 8.37% ± 0.036%			
				EE: 82.12% ± 1.13%			
	Liposomes incorporated into a commercial	Gentamycin Sulphate	Phosphatidylcholine (PC) from egg yolk	PS: 100 ± 19 nm	*In vitro*	Controlled and gradual release of antibiotic over a longer, 30-day period	(107)
	PMMA bone cement		Cholesterol			Enhanced the toughness, bending strength and Vickers hardness of the cement, without altering its polymerization or molecular structure	
			Pluronic L31, L61, and L43				
Inorganic Nanodelivery Systems	Nanohydroxyapatite drug complex pellets	Vancomycin	Nanohydroxyapatite		Rabbit model	Superior antibacterial activity in osteomyletic bone	
	Metallic Nanoparticles	NanoSilver cement	Silver	PS: 5–50 nm	*In vitro*	Improved performance when compared to gentamicin loaded cement	(112)
			PMMA				
	Silk fibroin nanoparticles		Silk	PS: 80–90 nm	Rat model	Improved Biocompatibility	(114)
				PDI: 0.100		Sustained release properties	
				ZP: −18 mV			
	Mesoporous Silica Nanoparticles	D_6_ peptide, UBI_29-41_ peptide, PEG-D_6_, and PEG-UBI_29-41_		EE: 19.00% ± 0.57% and 15.96% ± 0.14%	Rat model	Reduced Cytotoxicity	(115)
						Improved biocompatibility	
						Excellent bone-targeting	
	Borate bioactive glass (TBG)	Teicoplanin	Borate bioactive glass		Rabbit model	Improved drug delivery when compared to i.v. administration	(116)
						Borate glass has the advantages of better mechanical strength, more desirable kinetics of release of TEC, and a higher osteogenic capacity compared to calcium sulphate	
	Ontozeolitic imidazole frameworks (ZIFs)	Catechin		PS: 220 nm	*In vitro*	Biofilm reduction	(142)
				ZP: −7.61 mV			
	Nanofibrous scaffold	Vancomycin	poly-l-lactic acid		Rat model	Significant bacterial reduction	(117)
			Silicon				
			Nanohydroxyapatite				
						Potential for bifunctionality	
			gelatin				
	Silica nanoparticles	Chlorhexidine	tetraethyl orthosilicate (TEOS)	PS: 55.1 ± 8.3 nm	*In vitro*	Non-inferior performance of the bone cements containing chlorhexidine releasing silica nanocarriers to the equivalent commercial formulation	(120)
			3-aminopropyltriethoxysilane (APTS)				
			Sodium alginate				
						Superior antimicrobial activity against different bacterial species	
Polymeric Nanodrug Delivery Systems	lipid–polymer hybrid nanoparticle	Linezolid	1,2-Distearoyl-sn-glycero-3-phosphoethanolamine-*N*-[methoxy(polyethylene glycol)-2000] (DSPE-mPEG_2000_)	PS: 115.7 ± 0.4 nm	Rat model	Enhanced efficacy when compared to free payload	(124)
			Cholesterol	PDI: 0.16 ± 0.05			
			DPPE-lissamine rhodamine B (1,2)-dipalmitoyl-*sn*-glycero-3-phosphoethanolamine-*N*-(lissamine rhodamine B sulfonyl)	ZP: –43.0 ± 1.6 mV			
			lecithin				
			PLGA				
	Chitosan Nanoparticles	Antimicrobial peptides	Chitosan	PS: 121.13 ± 1.01 nm	*In vitro*	minimal toxicity against human erythrocytes	(125)
			Tripolyphosphate	ZP: 33.2 ± 2.6 mV			
				PDI: 0.272			
				EE: 51.33% ± 1.52%			
	Nanospheres	Vancomycin	PLGA		Rabbit model	No requirement for surgical removal	(126)
		Ceftazidime				Integration of infection control and pain relief	
		Lidocaine					
Stimuli-Responsive Nanomaterials	Gold nanoparticle-stabilized phospholipid liposomes	Vancomycin	Hydrogenated L-R-phosphatidylcholine (Egg PC)	PS: 110 ± 1 nm	*In vitro*	Toxin triggered release	(127)
			Cholesterol	ZP: –14.1 ± 0.4 mV		Reduced off-target drug delivery	
			Chitosan-50				
	Nanovalves		β-Cyclodextrin	PS: 850 nm		Reduced drug leakage	(141)
	Micelles	Chlorhexidine	polyvinyl caprolactam-polyvinyl acetate-polyethylene glycol graft copolymer	PS: 59.53 ± 0.2 nm; PDI: 0.09 ± 0.02	3D artificial dermis model	Improved chlorhexidine penetration	(128)
			Solutol HS 15	EE: 64.6 % ± 2.2 %		promoted maximum reduction in biofilm biomass	
						Enzymatic responsive drug delivery	
	Superparamagnetic iron oxide nanoparticles (SPIONs)		Iron		*In vitro*	Hyperthermia triggered drug delivery	(129)
		Gentamicin and Vancomycin	hyaluronic acid		Rabbit and Sheep	Outperforming clinically available alternatives	(130, 131)
	Carbon dot-based nanocomposites		Lysine		*In vitro*	pH-responsive drug delivery	(132)
			mPEG-PEI-DMMA				
	Titanium Nanotubes	Antimicrobial peptides	poly(methacrylic acid)		*In vitro*	Bacterial infection responsive drug delivery	(133)
						pH-responsive drug delivery	
						Excellent bactericidal activity	
	Gold Nanoparticles		(10-mercaptodecyl) trimethylammonium bromide	PS: 14 nm	Rabbit	NIR conversion bactericidal	(134)
			11-mercaptoundecanoic acid			pH responsive drug delivery	
	Iron oxide Nanoparticles			PS: 8, 11, and 70 nm	*In vitro*	Magnetic field directed drug delivery	(135)
	Nano cyclodextrin supramolecular complex		Cyclodextrin	PS: 142.1 ± 3.1 nm	Mouse	pH responsive drug delivery	(136)
			poly(ethylene glycol)			nitric oxide producing bactericidal activity	
						Reactive Nitrogen Species generation bactericidal activity	
	Mesoporous silica nanoparticles		Hexadecyltrimethylammonium bromide (CTAB)	PS: 2–10 nm	Rat	Enzyme-responsive drug release	(137)
			Tetraethylorthosilicate (TEOS)				
			*N*-(aminoethyl)-amino-propyl trimethoxysilane				

## 5 Future Perspectives

The use of nanomaterials for the treatment of medical conditions has recently gained more traction in the face of new pandemics. Exploring ways of manipulating nanomaterials to help treat infections that do not respond to standard medical treatment could potentially offer a range of new options for combating antimicrobial resistance (AMR). Although each functionalization/modulation method for nanomaterials has been individually suggested for the treatment of AMR, it is also necessary to take into account how to deliver the cutting-edge technology to the organ or tissue infected. In light of this, scientists have realized that the combination of modulation and targeted drug delivery may result in a novel platform with additional properties and improved performance, particularly in the management of infections that are difficult to treat, like BJIs.

When utilized in the management of BJIs, nanomaterials have the ability to penetrate bacterial biofilms and directly target bacteria more effectively than traditional medicine. The possibility of loading them with antibiotics can be explored, which will allow for sustained release and targeted delivery to the site of infection. Overall, the use of nanomaterials in the management of BJIs shows great potential for improving treatment outcomes and therefore helping in the reduction of AMR.

Nevertheless, although nanomaterials hold a lot of potential, there are some limitations that need to be considered, specifically with BJIs. Bone penetration to the areas within which BJI occur is considerably harder and in many cases requires surgical interventions. The primary strategy for combating this issue is based on the design, development, and successful fabrication of nanomaterials that can deposit the payload of antimicrobials in bone tissue without the need for surgery. This still presents a significant challenge because bone and joint tissue are deeply entrenched in the human. Although direct injection may still be necessary in some situations, it appears that the use of stimuli-responsive nanomaterials has the greatest potential to overcome some of these difficulties.

An interesting and possibly revolutionizing approach would be to utilize bone targeting strategies. Utilizing strategies including in active and passive bone targeting, bone targeted biomaterials, and bone-targeted moieties in conjunction with nanotechnology has demonstrated significant success,^143^ and extrapolating these strategies for antimicrobial resistant species opens a world of possibilities.

The boundaries of these alternative drug delivery platforms and their application to BJIs must be defined because this novel field of research is both very exciting and still in its infancy. It is likely that scientists will test a wide range of different nanomaterials in the upcoming years including ethosomes/niosomes/liposomes, metallic NPs, and silica NPs and possibly incorporate nanofiber enforced hydrogels among many others, either separately or in combination. These can be developed to defeat both AMR and surgical procedures simultaneously.

Moreover, a more additive combination would be the application of bone targeted cell-derived biomimetic nanomaterials to improve bone deposition without surgery. However, the effects and limits regarding the possibility of combining different techniques as well as potentially adding biomimetics would need to be defined.

A particular area of concern regarding this exciting field of nanomaterials in AMR and BJIs would be possible negative consequences resulting from organisms subsequently developing resistance to these new technologies, which will be challenging to overcome taking into account the complexity of the field.

This research field should certainly be explored, and it is definitely going to bring a substantial number of new ideas for biomedical and drug delivery applications.

## 6 Conclusions

BJIs have long been a concern particularly among pediatrics and the elderly. The increase in the life expectancy among people may translate into an increase in the prevalence of surgeries such as hip replacements that leave patients susceptible to infections. Of concern, however, is the increasing occurrence of superbugs developing mechanisms of circumventing the pharmacological effects of commonly used antibiotics. The use of nanotechnology has been demonstrated to be able to provide a new lease of life to already developed medicines against theses superbugs. While this paper highlights the successful use of nanotechnology in resistant bacteria for BJIs, there appears to be a dearth of work to be explored for its use in the treatment of resistant organisms causing BJIs and other tissue infections. It remains imperative to develop novel drug delivery systems to improve the outcomes of microbial treatment for BJIs and bacterial infections in general.
